# Extracellular Vesicles from *Candida haemulonii* var. *vulnera* Modulate Macrophage Oxidative Burst

**DOI:** 10.3390/jof9050562

**Published:** 2023-05-12

**Authors:** Bianca T. M. Oliveira, Thales M. H. Dourado, Patrick W. S. Santos, Tamires A. Bitencourt, Carlos R. Tirapelli, Arnaldo L. Colombo, Fausto Almeida

**Affiliations:** 1 Department of Biochemistry and Immunology, Ribeirão Preto Medical School, University of São Paulo, Ribeirão Preto 14049-900, SP, Brazil; 2 Department of Pharmacology, Ribeirão Preto Medical School, University of São Paulo, Ribeirão Preto 14049-900, SP, Brazil; 3 Laboratory of Pharmacology, Department of Psychiatric Nursing and Human Sciences, College of Nursing of Ribeirão Preto, University of São Paulo, Ribeirão Preto 14040-902, SP, Brazil; 4 Special Laboratory of Mycology, Universidade Federal de São Paulo, São Paulo 04023-062, SP, Brazil

**Keywords:** *Candida haemulonii* species complex, fungal extracellular vesicles, oxidative stress, immunomodulatory activity

## Abstract

Members of the *Candida haemulonii* species complex are multidrug-resistant emergent yeast pathogens able to cause superficial and invasive infections in risk populations. Fungal extracellular vesicles (EVs) play a critical role in the pathogenicity and virulence of several species and may perform essential functions during infections, such as carrying virulence factors that behave in two-way communications with the host, affecting survival and fungal resistance. Our study aimed to describe EV production from *Candida haemulonii* var. *vulnera* and evaluate whether murine macrophage RAW 264.7 cells respond to their stimuli by generating an oxidative response after 24 h. For this purpose, reactive oxygen species detection assays demonstrated that high concentrations of yeast and EVs (10^10^ particles/mL) of *Candida haemulonii* did not change macrophage viability. However, the macrophages recognized these EVs and triggered an oxidative response through the classical NOX-2 pathway, increasing O_2_^•−^ and H_2_O_2_ levels. However, this stress did not cause lipid peroxidation in the RAW 264.7 cells and neither lead to the activation of the COX-2–PGE_2_ pathway. Thus, our data suggest that low concentrations of *C. haemulonii* EVs are not recognized by the classical pathway of the oxidative burst generated by macrophages, which might be an advantage allowing the transport of virulence factors via EVs, not identified by the host immune system that could work as fine tube regulators during infections caused by *C. haemulonii*. In contrast, *C. haemulonii* var. *vulnera* and high EV concentrations activated microbicidal actions in macrophages. Therefore, we propose that EVs could participate in the virulence of the species and that these particles could be a source of antigens to be exploited as new therapeutic targets.

## 1. Introduction

Infections caused by *Candida* spp. are a prone threshold for public health and have emerged as a challenge for proper diagnosis due to their varied virulence profiles [1,2,3,4]. *Candida haemulonii* is a multifaceted group of species formed by *C. haemulonii sensu stricto*, *C. haemulonii* var. *vulnera*, *C. vulturna*, and *C. duobushaemulonii.* These species represent a challenge to correctly identify and treat, owing to their multidrug resistance profile [5,6,7,8]. *C. haemulonii* var. *vulnera* can cause diseases with different clinical manifestations, from superficial to deep infections, especially in newborns, patients with diabetes, and critically ill patients exposed to invasive medical procedures and antibiotics, in addition to immunocompromised patients [9,10,11,12]. Their invasiveness could be partially explained by the main virulence factors of this genus, such as the ability to produce extracellular enzymes, such as phospholipases and hemolysin, during candidemia [13,14], formation of biofilms [15], induction of stress responses, acquired multiple mutations in drug efflux pumps, and expression of genes involved in ergosterol biosynthesis [16,17], and release of EVs [18,19].

EVs are lipid-bilayer structures released by all organisms [20,21]. Fungal EVs carry many biologically active molecules, including proteins, nucleic acids, lipids, pigments, toxins, gene regulators, and virulence factors [22,23,24,25]. Thus, they are considered an alternative system for intercellular communication and play essential roles in microbial structure and pathogenesis during host–pathogen interactions [26,27,28,29]. Moreover, fungal EVs modulate the activation of the innate immunity [30,31]. Several in vitro studies have demonstrated that fungal EVs can activate immune responses in effector cells, such as macrophages and neutrophils, modulating phagocytosis, affecting macrophage polarization to M1 or M2, increasing chemokine and cytokine levels, and stimulating the production of reactive oxygen species (ROS) [32,33,34,35,36,37].

During infections, the balance between ROS production and the fungal stress response is the central axis of the host–pathogen interaction and can define the outcome of the fungal infection. The host response mechanisms include formation of superoxide (O_2_^•−^), hydrogen peroxide (H_2_O_2_), and nitric oxide (NO) [38,39,40,41]. They participate in cellular systems and processes critical for the microbicidal action of macrophages [42,43]. Furthermore, inflammatory mediators, such as prostaglandins (PGs), are secreted in high quantities by macrophages, acting as autocrine modulators and regulating cytokine production by activated macrophages [44,45,46,47,48].

In the present study, we describe for the first time that *Candida haemulonii* produces EVs. Our findings provide experimental evidence for the recognition by murine macrophages of these fungi species and their EVs. Taken together, our results suggest new insights in the pathophysiology of *C. haemulonii* spp., demonstrating important host–pathogen interactions and providing new therapeutic targets.

## 2. Materials and Methods

### 2.1. Fungal Strains and Growth Conditions

The strain *Candida haemulonii* var. *vulnera* ATCC 1112 was grown at 30–37 °C in Sabouraud Dextrose medium (dextrose 40 g/L, peptone 10 g/L, agar 20 g/L) Oxoid, Basingstoke, UK) at pH 5.6 for 48 h [49]. Four fresh colonies were inoculated into 5 mL of Sabouraud broth and cultured at 30 °C with shaking (150 rpm). Subsequently, an EV isolation experiment was performed.

### 2.2. Production and Purification of EVs and Nanoparticle Tracking Analysis (NTA)

*C. haemulonii* EVs were isolated according to the methodology described by Vallejo et al. for *Paracoccidioides brasiliensis* [50]. For EV isolation, cells and debris were removed by sequential centrifugation at 5000× *g* for 15 min and 15,000× *g* for 15 min. The supernatants were concentrated using an Amicon ultra-concentration system (cutoff 100 kDa; Millipore, Billerica, MA, USA). The resulting supernatant was filtered through a 0.45 µm filter (Sigma-Aldrich, St. Louis, MO, USA). The resulting concentrated supernatant was ultracentrifuged at 100,000× *g* at 4 °C for 1 h. Pellets were collected and resuspended in ultra-pure water (Sigma-Aldrich) supplemented with protease inhibitor cocktail 10× (Sigma-Aldrich) (0.2% *v*/*v*) and stored at −80 °C. The size and distribution of the particles were evaluated using Nanoparticle Tracking Analysis (NTA) (NanoSight appliance NS300, Malvern Instruments, Worcestershire, UK) with NTA 3.0. The parameters were set according to the manufacturer’s instructions. The camera level was increased to >14 at which all particles were distinctly visible, and the threshold was determined to capture as many particles as possible within an ideal range of 20 to 100 particles per frame [32].

### 2.3. Electron Transmission Microscopy (TEM) Images

The pellets obtained from six independent preparations were fixed with glutaraldehyde 2.5% (*v*/*v*) + 4% (*v*/*v*) formaldehyde in sodium cacodylate buffer (0.1 M) at pH 7.2. Next, the samples were washed in PBS, incubated for 60 min in 1% osmium tetroxide (*v*/*v*), dehydrated in an ethanol series, and embedded in Spurr’s resin. Ultrathin sections (70 nm) were obtained using a Leica UC7 ultramicrotome (Leica Microsystems, Wetzlar, Germany) and contrasted using 5% (*w*/*v*) uranyl acetate for 20 min and 0.5% (*w*/*v*) lead citrate for 5 min. The samples were observed using a JEOL 1200EX transmission electron microscope operating at 80 kV [51,52].

### 2.4. Cell Culture

RAW 264.7 murine macrophages (ATCC, Manassas, VA, USA) were maintained in Dulbecco’s minimal essential medium (DMEM; Gibco™, Carlsbad, CA, USA) supplemented with 10% fetal bovine serum (FBS; Gibco™), 4 mM glutamine, and 1% penicillin-streptomycin (Gibco™) at 37 °C in a 5% CO_2_, humidified atmosphere. For all biochemical analyses, DMEM supplemented with 10% FBS exosome-depleted was used according to a previously described protocol [53]. RAW 264.7 cells are particularly sensitive to lipopolysaccharide (LPS). For the positive control, we first assessed RAW 264.7 cell viability in the presence of 1 µg/mL LPS (Sigma-Aldrich) that can be used to activate them [54].

### 2.5. Cell Viability Assay

RAW 264.7 cell viability after challenge with different concentrations of EVs from *C. haemulonii* was evaluated using the AlamarBlue^®^ (Sigma-Aldrich) assay to assess their cytotoxic effects. A total of 1 × 10^5^ cells per well were seeded into 96-well microplates for 24 h to allow for adhesion. After 24 h of exposure to EVs, 20 μL of AlamarBlue^®^ was added to each well and the plate was incubated again (4 h/37 °C/5% CO_2_). The reduction of resazurin was assayed at 570 nm and 600 nm using a microplate ELISA reader (iMark™, Microplate Absorbance Reader, Bio-Rad Laboratories, Hercules, CA, USA). DMEM was used as the negative control. All assays were performed in triplicate [55].

### 2.6. Measurement of ROS and NO Production

Three fluorescent probes, 2′,7′-dichlorodihydrofluorescein diacetate (H_2_DCFDA), dihydroethidium (DHE), and 4-amino-5-methylamino-2′,7′-difluorofluorescein diacetate (DAF), were used to measure ROS, O_2_^•−^, and NO production, respectively. Macrophages were seeded into black 96-well plates at a density of 1 × 10^5^ cells/well. After 24 h, the cells were washed twice with sterile PBS and incubated at 37 °C for 30 min with 10 µM H_2_DCFH-DA, 5 µM DHE, or 10 µM DAF for 45 min. The fluorescence was measured using a microplate reader (iMark™, Microplate Absorbance Reader) (λEx 485 nm, λEm 528 nm for DCFH-DA [56], λEx 540 nm, λEm 590 nm for DHE [57], and λEx 495 nm, λEm 515 nm for DAF) [58] and results are expressed as Relative Fluorescence Units (RFU).

### 2.7. Western Immunoblotting

The expression of gp91-phox (NOX-2) and cyclooxygenase-2 (COX-2) proteins in RAW 264.7 cells was analyzed using the western immunoblotting assay [59]. After incubation, the cells were collected, washed with ice-cold phosphate-buffered saline (PBS), and lysed in ice-cold RIPA buffer (50 mM Tris HCl, pH 7.4, 150 mM NaCl, 1 mM EDTA, 0,.1% SDS (*w*/*v*), 1% Triton X-100 (*v*/*v*), 1.5 mM diethyl tritol (DTT), Protease and Phosphatase Inhibitor Cocktail (Sigma-Aldrich)). The protein concentration of the cell lysates was estimated using Bio-Rad Dc reagents for protein assay according to the manufacturer’s instructions (Bio-Rad Laboratories), and bovine serum albumin (BSA) was used as the standard. Thirty micrograms of each protein sample were separated on an SDS-PAGE gel and transferred onto a nitrocellulose membrane (Millipore, Burlington, MA, USA). The membrane was blocked with 7% skimmed milk (Molico–Nestle^®^, Vevey, Switzerland) in Tris-buffered saline with Tween 0.1% (*v*/*v*) (TBS-T) for 1 h. After blocking, the membranes were incubated at 4 °C overnight with one of the following primary antibodies: Anti-gp91-phox Mouse monoclonal (1/500, sc-130543, Santa Cruz Biotechnology, Santa Cruz, CA, USA) and Anti-Cox-2 Mouse Polyclonal (1/500, 160126, Cayman, Chemical Company, Ann Arbor, MI, USA). After incubation with the primary antibodies, the membranes were washed three times in TBS-T and incubated with secondary antibodies at room temperature for 90 min. The signals were detected using a chemiluminescent reagent and visualized using ChemiDoc XRS+ (Bio-Rad). The proteins were quantified using densitometry (Image Lab software 6.1, Bio-Rad), and β-actin (1:500, sc-4778, Santa Cruz Biotechnology) was used as the internal control. Image Lab software uses a regression method to calculate the molecular weight of protein bands. The area of interest was the same for all other bands, 26.8 mm^2^. The background was subtracted of the volume of each band. The band quantification was calculated as the ratio of the background-adjusted volume sample divided by the background-adjusted beta-actin volume.

### 2.8. Measurement of Prostaglandin E_2_

The supernatants from cells stimulated with EVs and challenged with *C. haemulonii* were obtained, and the levels of prostaglandin E_2_ (PGE_2_) were measured by ELISA. PGE_2_ concentration released into the culture medium was quantified using a specific enzyme immunoassay (EIA Kit) according to the manufacturer’s instructions (514010, Cayman). The results for PGE_2_ concentrations are expressed in pg/mg.

### 2.9. Malondialdehyde Concentration

The cells were incubated in 96-well plates at 1 × 10^5^ cells/well for 24 h in all groups (macrophages; *C. haemulonii* and 10^5^ to 10^10^ fungal EVs). Culture supernatants (100 μL) were collected in microcentrifuge tubes and Milli-Q^®^ water (Merck Millipore, Burlington, MA, USA) (100 μL), 8.1% sodium dodecyl sulfate (SDS) (100 μL), acetic acid buffer pH 3.5 (350 μL), and 0.6% thiobarbituric acid (350 μL) were added to each tube. A standard curve of malondialdehyde (MDA) was prepared using concentrations of 22.5, 15, 7.5, 3.75, and 0 mol/L. The samples were then left in a heated bath (95 °C/1 h) and centrifuged (1600× *g*, 10 min, 4 °C). The supernatant (150 µL) was transferred into a 96-well plate and the concentration of MDA was determined colorimetrically (532 nm) using the standard curve for MDA as a reference. The results are expressed in mmol/mL [60].

### 2.10. Statistical Analysis

All experiments were performed in triplicate in three independent experimental sets. Statistical analyses were performed using GraphPad Prism version 8.0.1. The results are presented as the mean ± standard error of the mean (SEM). One-way analysis of variance (ANOVA) followed by the Bonferroni post-test was performed to detect differences between the values under study. Statistical significance was set at * *p* < 0.05.

## 3. Results

### 3.1. EV Size and Distribution

**Figure 1:**
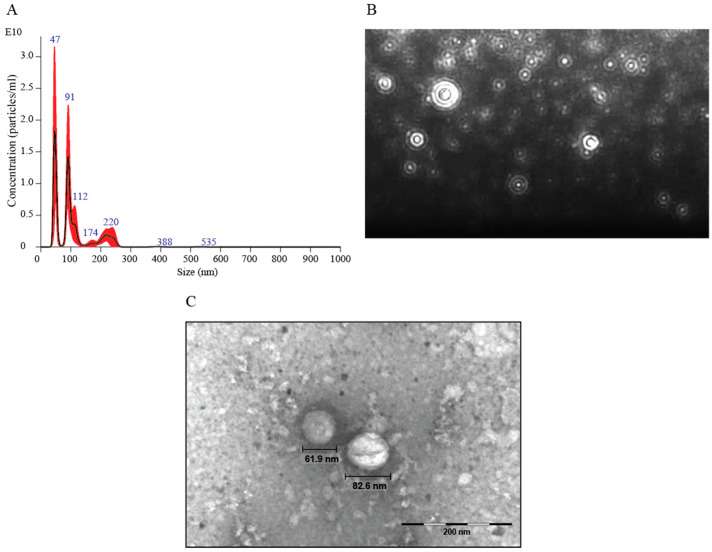
Extracellular vesicles (EVs) produced by *Candida haemulonii* var. *vulnera*. (**A**) Nanoparticle-tracking analysis of EVs isolated from *C. haemulonii* culture supernatant was performed using NanoSight NS300. Representative graph depicting the particle-size distribution and concentration of EV profiles from *C. haemulonii* (EVs × 10^11^ particles/mL). (**B**) Screenshot from the video recorded using NanoSight NS300, presenting EV distribution. (**C**) Image produced using transmission electron microscopy (TEM). The EVs from *C. haemulonii* showed a globose morphology and the diameter of these EVs was in the range of the diameters of EVs obtained using Nanoparticle Tracking Analysis (NTA).

The size and distribution profiles of *C. haemulonii* var. *vulnera* EVs were determined using NTA, as shown in Figure 1. These EVs ranged in size from 60 to 150 nm, and the average size of those obtained from several cultures was 131.6 nm with a size peak at 102.7 nm (Figure 1A). The size and distribution profiles of these EVs were screened from a video recorded using a NanoSight NS300 system (Malvern Instruments, Worcestershire, UK) (Figure 1B). Transmission electron microscopy (TEM) analysis revealed the presence of spherical structures delimited by electrodense bilayers characteristic of EVs (Figure 1C). Quantification showed that the isolated EVs had the same average diameter as those demonstrated using NTA.

### 3.2. Viability Assay

**Figure 2:**
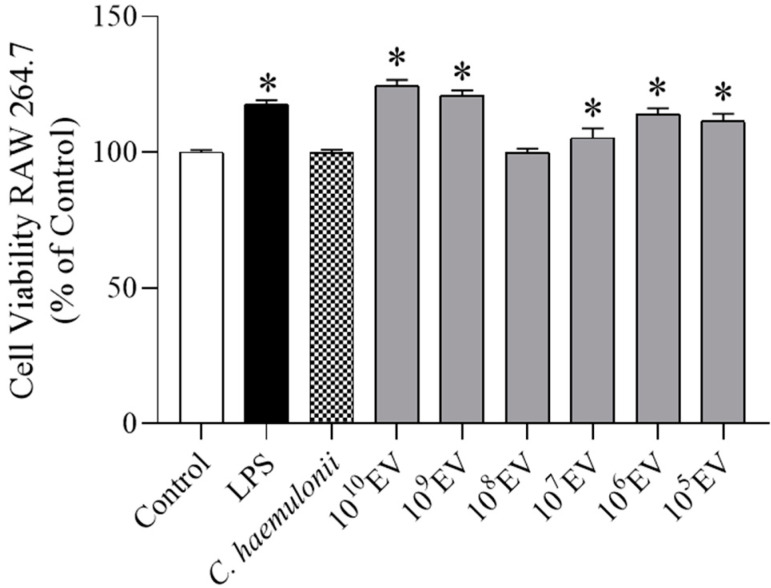
Viability of RAW 264.7 cells stimulated with increasing concentrations of extracellular vesicles (EVs) from *Candida haemulonii* var. *vulnera*. RAW 264.7 cells were incubated for 24 h with increasing concentrations of EVs from *C. haemulonii* var. *vulnera*. The viability of RAW 264.7 cells incubated without EVs, lipopolysaccharide (LPS), or *C. haemulonii* was defined as 100% (Control). Data are expressed as the mean ± SEM of three independent experiments performed in triplicate, * *p* < 0.05 vs. control (one-way ANOVA, followed by Bonferroni test).

The cytotoxicity of *C. haemulonii* EVs was assessed by measuring the metabolic activity of RAW 264.7 cells using the AlamarBlue^®^ assay (Figure 2). Cell viability was measured after exposure to increasing EV concentrations ranging from 10^10^ to 10^5^ particles/mL or 1 µg/mL LPS (positive control) and *C. haemulonii* for 24 h. The results showed that there was an increase in cellular metabolism and no changes in cell viability.

### 3.3. C. haemulonii EVs Increase ROS Production in RAW 264.7 Macrophages

**Figure 3:**
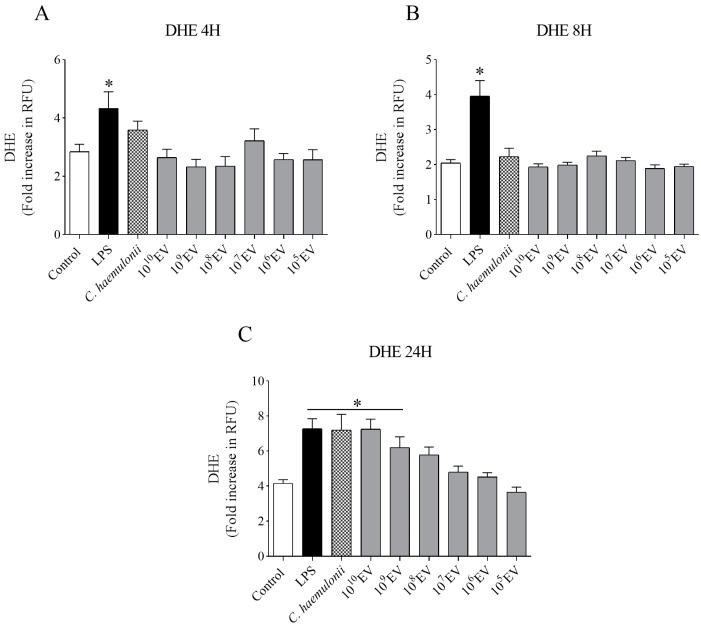
Measurement of reactive oxygen species (ROS) production in RAW 264.7 cells stimulated with *Candida haemulonii* and increasing concentrations of their extracellular vesicles (EVs). Superoxide anion concentration after 4 h (**A**), 8 h (**B**), and 24 h (**C**) of incubation was determined by measuring the fluorescence of the dihydroethidium (DHE) probe (λEx 540 nm, λEm 590 nm). LPS: lipopolysaccharide. Data are expressed as the mean ± SEM of three independent experiments performed in triplicate, * *p* < 0.05 vs. control (one-way ANOVA, followed by Bonferroni).

For *C. haemulonii* var. *vulnera* ATCC 1112 and its EV preparations (10^10^–10^5^ particles/mL) incubated in macrophage culture, we found that only higher EV concentrations (10^10^ and 10^9^ particles/mL) significantly increased superoxide anion production in RAW 264.7 macrophages (* *p* < 0.05) (Figure 3). After obtaining the DHE probe results, we used the highest concentrations (10^10^ and 10^9^ particles/mL) for 24 h as the conditions for further experiments.

### 3.4. C. haemulonii EVs Increase NOX-2 Expression and H_2_O_2_ Levels

**Figure 4:**
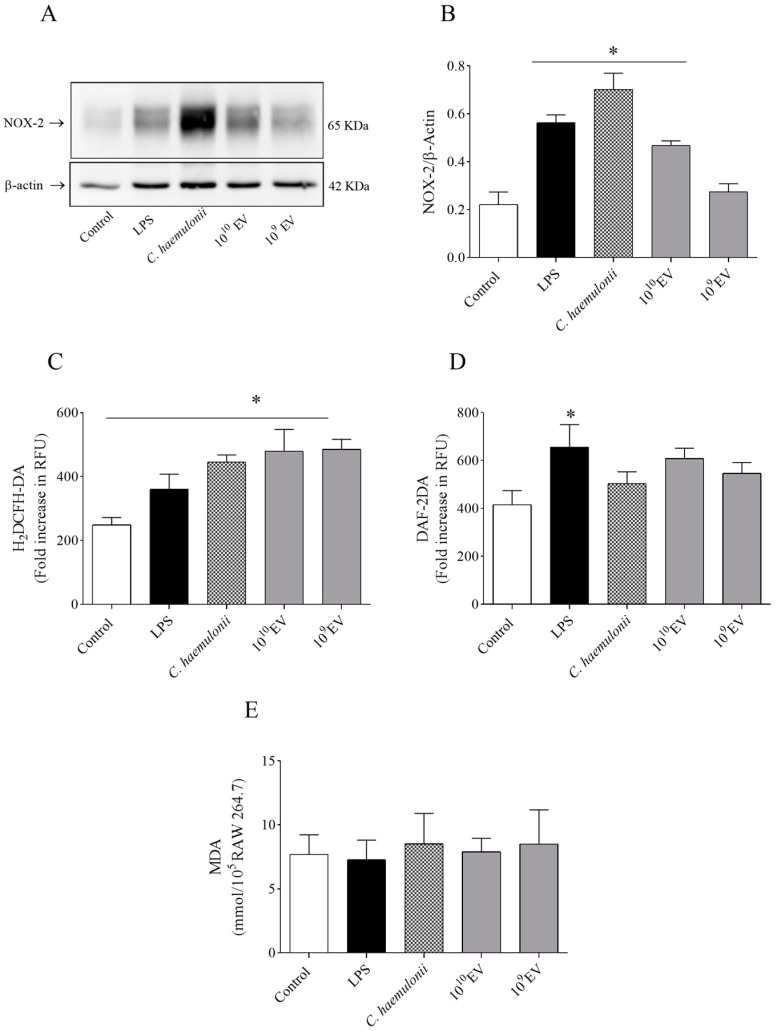
*Candida haemulonii* var. *vulnera* extracellular vesicles (EVs) induce an increase in reactive oxygen species (ROS) levels. (**A**) Representative immunoblot for NOX-2 (gp91-phox). (**B**) Representative bar graph for NOX-2. Immunoblot results are shown as the mean ± SEM of *n* = 6. (**C**) After a 24 h incubation, *C. haemulonii* and its EVs (10^10^–10^9^ particles/mL) increased H_2_O_2_ levels in RAW 264.7 cells. ROS production was determined by measuring the fluorescence of the 2′,7′-dichlorodihydrofluorescein diacetate (H_2_DCFH-DA) probe (λEx 485 nm, λEm 528 nm). (**D**) Nitric oxide levels were measured using the fluorescent probe 4-amino-5-methylamino-2′,7′-difluorofluorescein diacetate (DAF) (λEx 495 nm, λEm 515 nm). (**E**) Malondialdehyde (MDA) concentration in RAW 264.7 cells after 24 h exposure to all stimuli. We demonstrate that oxidative bursts generated from these groups were not harmful to macrophages. LPS: lipopolysaccharide. Data are expressed as the mean ± SEM of three independent experiments performed in triplicate, * *p* < 0.05 vs. control (one-way ANOVA, followed by Bonferroni test).

Our results showed an increase in NOX-2 expression in macrophages challenged with LPS, *C. haemulonii*, or high EV concentrations (Figure 4A). Furthermore, our data showed an increase in fluorescence for the H_2_DCFH-DA probe, indicating a boost in H_2_O_2_ levels in all groups (Figure 4B). However, only the LPS group showed increased fluorescence of the DAF probe (Figure 4C). There was no statistical difference in the MDA concentrations between the groups (Figure 4D).

### 3.5. Evaluation of Prostaglandin E_2_ Levels and Cyclooxygenase-2 Expression

**Figure 5:**
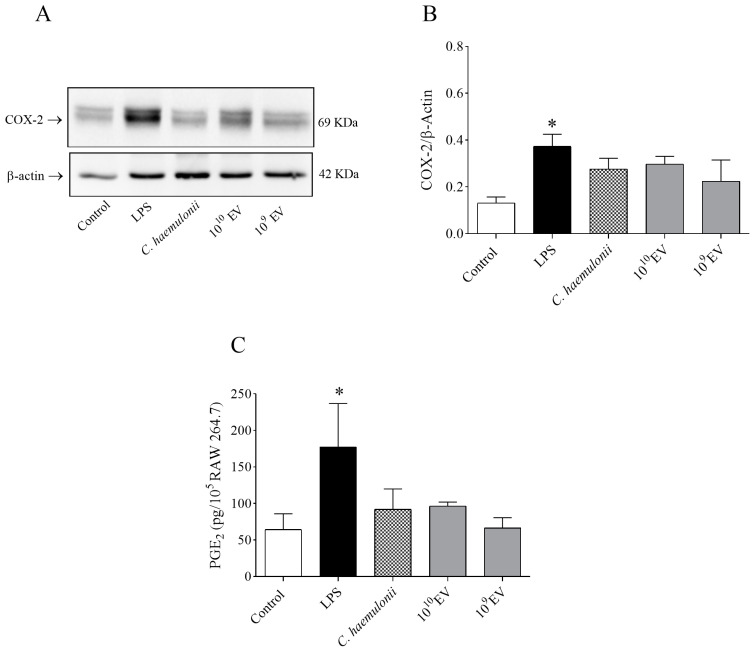
*Candida haemulonii* var. *vulnera* extracellular vesicles (EVs) did not induce an increase in the expression levels of PGE_2_ and COX-2. (**A**) Representative immunoblot for COX-2. (**B**) Representative bar graph for COX-2. (**C**) PGE_2_ levels determined using ELISA. LPS: lipopolysaccharide. Results are shown as the mean ± SEM of *n* = 6. One-way ANOVA followed by Bonferroni’s multiple comparison test were used to compare groups (* *p* < 0.05).

Our ELISA and Western blot results showed that there was no difference in COX-2 (Figure 5A,B) and PGE_2_ (Figure 5C) levels, respectively, in RAW 264.7 cells treated with *C. haemulonii* var. *vulnera* for 24 h. COX-2 and PGE_2_ levels were only found to be increased following treatment with LPS (positive control).

## 4. Discussion

The *Candida haemulonii* species complex encompass pathogenic yeasts that are all considered phylogenetic relatives of *C. auris*. They have emerged as dangerous opportunistic pathogens in hospitals owing to their resistance to multiple antifungal agents and phenotypic similarity to other *Candida* strains [49,61,62,63,64,65]. This challenging scenario highlights the relevance of characterizing virulence factors that may potentially support effective treatment against essential targets during diseases, such as undiscovered machinery for producing fungal EVs. These particles are considered critical carriers of several antigenic biomolecules during infections [50,52,66].

Fungal EVs participate in the immunomodulation of the host and pathogenicity of species [31,67,68]. EVs from *Cryptococcus neoformans* are phagocytosed by macrophages, inducing cell activation and NO and cytokine production [36], whereas *C. albicans* EVs activate RAW 264.7 cells resulting in NO production and release of IL-12, IL-10, TGF-β, and TNF-α [69]. Another example of EV interaction with the immune host system is EVs released from *Trichophyton interdigitale*, which stimulate the release of NO, TNF-α, IL-6, and IL-1β, but not IL-10, from murine macrophages and human keratinocytes in a dose-dependent manner [37].

However, to our knowledge, the occurrence of EVs from *C. haemulonii* group of species is unprecedented, and their functions and composition remain unknown. To characterize these EVs, we evaluated the size, morphology, and recognition of a population of EVs from this pathogenic fungus by RAW 264.7 cells. To elucidate the infection pathway and establish its influence in a mammalian cell model, the dimensions of EVs can be related to specialized functions in their cellular composition [70,71].

Our results demonstrate that EVs comprised a heterogeneous population 60–150 nm in size with a small subpopulation of 200 nm. The profiles are similar to the patterns observed in previous studies for species belonging to the same clade as *C. auris* [35], the same genus as *C. albicans* [69], and yeasts of a different genus, such as *C. gattii* [72]. Furthermore, visualization of these EVs using TEM demonstrated a spherical and globose morphology that was slightly flattened, with dimensions between 60 and 140 nm. These EV characteristics are consistent with those of EVs from *C. albicans* and *C. auris* [35].

Previous in vitro studies have shown potential cytotoxic effects of fungi, compromising macrophage viability during co-incubation. For example, *Trichophyton rubrum* conidia and *C. albicans* yeast differentiated into hyphae whose populations were increased and induced lysis of RAW 264.7 cells after co-incubation for 8 h [73,74,75,76]. In this study, we demonstrated that *C. haemulonii* var. *vulnera* or its EVs did not exhibit cytotoxicity against RAW 264.7 macrophages. On the contrary, these results suggest that the cellular metabolism of these cells was stimulated by the high percentage conversion of resazurin to resofurin in the AlamarBlue assay. In support of our data, previous studies showed that *C. neoformans* and *C. albicans* EVs did not modify macrophage viability but could stimulate their functions, mainly by activating their antimicrobial activity [36,69,77,78].

Oxidative burst is a macrophage response to fungal infections. The oxidative molecules produced participate in phagocytosis, fungal killing, and the regulation of physiological processes regarding host immunity [74,79,80,81,82,83]. Thus, we evaluated ROS and NO production using three fluorescent probes: DHE, H_2_DCFH-DA, and DAF. Thereby, we investigated the ability of macrophages to recognize, become activated, and produce ROS following stimulation with *C. haemulonii* var. *vulnera* and its EVs. The results obtained using DHE indicated a concentration-dependent increase in ROS after 24 h of incubation with *C. haemulonii* EVs. Therefore, we focused only on the high concentrations of EVs (10^10^ and 10^9^ particles/mL) for the subsequent experiments.

The DHE and H_2_DCFH-DA probes are sensitive to ROS, such as O_2_^•−^ and H_2_O_2_, whereas the DAF probe responds to NO [54]. Our data suggest that RAW 264.7 macrophages did not effectively recognize *C. haemulonii* EVs at low concentrations or incubation times shorter than 24 h. Thus, the production of ROS and NO by *C. haemulonii* is rate limiting and can help the yeast adapt and colonize the host. Other studies corroborate these hypotheses, demonstrating the failure in recognizing *Candida sp*. cell wall variations, such as singular mannan content with different β-1,2-linkages [84]. The reduced ability of *Candida* sp. to induce oxidative stress damage caused by phagocytes has also been reported [85,86]. In contrast, the production of NO by murine macrophages stimulated by *C. neoformans* or *C. albicans* EVs is concentration dependent [36,69]. However, under the experimental conditions of this study, there was no significant difference in NO production following stimulation with *C. haemulonii* EVs, even at high concentrations.

ROS in phagosomes damage the fungal cell membrane by causing redox imbalance in the invading pathogen [77,78]. ROS generated by the NADPH-oxidase complex are highly regulated by diverse microenvironmental factors, and NOX-2 is responsible for the oxidative bursts generated against fungal infections [87,88]. Previous in vitro assays demonstrated the microorganism’s ability to harm NOX-2 by interacting with its subunits, or modulate cellular processes through EVs, as displayed by *Histoplasma capsulatum* EVs, in which protein inhibition was associated with a decrease in ROS production by macrophages [89].

Furthermore, we evaluated whether gp91-phox mediated the oxidative burst caused by *C. haemulonii* var. *vulnera* and its EVs. Since this isoform is prevalent in macrophages, a western immunoblotting assay demonstrated increased expression of NOX-2 in this fungal species [42,90]. We hypothesized that classical macrophage activation is critical for recognizing and eliminating the *C. haemulonii* group of species. For example, in *C. albicans* infection, NOX-2 promotes phagocyte chemotaxis and intracellular fungal containment restricts hyphae growth [91]. In alveolar macrophages, NOX-2 activation is essential for killing *Aspergillus* sp. spores and maintaining homeostasis [92].

ROS perform essential functions during infection. However, overproduction of ROS damages phagocytes via lipid peroxidation [93,94]. *C. neoformans*, *C. albicans*, and *Aspergillus fumigatus* strains increased malondialdehyde (MDA) levels in alveolar macrophages suggesting lipid peroxidation during fungal challenge [95,96]. Our results showed no difference in MDA levels in RAW 264.7 macrophages stimulated with *C. haemulonii* yeast or its EVs, suggesting that ROS damage against this species was compartmentalized in RAW 264.7 cells.

Cyclooxygenases (especially isoform 2, COX-2) catalyze the stage-limiting step in the synthesis of PGs and thromboxane [44,45]. Previous studies have demonstrated that during infections, activated macrophages secrete prostaglandin E_2_ by inducing cyclooxygenase-2 expression [88,97]. These molecules participate in acute inflammation by inducing phagocytosis and cell proliferation [98]. Moreover, during *C. albicans* infections, upregulation of COX-2 and PGE_2_ signaling stimulates Th2 and Th17 responses to yeast and limits the ability of macrophages to kill *Candida sp.* [99,100].

We analyzed whether 24 h of incubation with *C. haemulonii* var. *vulnera* and its EVs stimulated COX-2 expression and PGE_2_ production in RAW 264.7 cells. Our results indicated that the COX-2–PGE_2_ signaling pathway was not activated by the yeast or its EVs. Previous reports have shown that *C. albicans* mannans are fungal components that directly induce PGE_2_ production [100]. We hypothesized that the composition of the *C. haemulonii* cell wall might be different and not effectively recognized by macrophages to activate this inflammatory pathway, which awaits further investigation into whether another signaling pathway could be active during this kind of infection [84,99].

Taken together, our data showed that *C. haemulonii* can produce and release EVs. These particles are immunogenic and stimulate microbicidal function in murine macrophages by inducing ROS generation. Our findings may widen the knowledge about *C. haemulonii* infection and the role of EVs in the immunomodulatory landscape.

We suggest *C. haemulonii* EVs deliver critical biomolecules for the virulence of this species. At high concentrations, they can be recognized and activate the host immune response owing to the presence of particular antigens in these species. Therefore, fungal EVs could be used as new therapeutic targets for efficient vaccines or antifungals. Meanwhile, low concentrations of EVs do not activate the oxidative burst, facilitate bidirectional communication of the fungus, and transport virulence factors for fungal colonization.

## 5. Conclusions

Our data demonstrate that *C. haemulonii* var. *vulnera* produce EVs, particularly those with small diameters. Macrophages recognize these EVs, although no cytotoxicity or induced lipid peroxidation was observed. These EVs stimulated ROS production by increasing NOX-2 activity. However, the COX-2–PGE_2_ pathway was not stimulated after 24 h of EV exposure. Further studies may elucidate the critical functions in virulence and pathogenicity and the content of *C. haemulonii* var. *vulnera* EVs.

## Data Availability

The raw data supporting the conclusions of this article will be made available by the authors, without undue reservation.
